# Engineered *Bacillus subtilis* for high-yield nattokinase production and therapeutic potential in obesity management

**DOI:** 10.1186/s40104-026-01438-3

**Published:** 2026-06-23

**Authors:** Yilin Liu, Lei Zhu, Tingyu Yao, Yanfeng Wang, Chunyan Xie, Le Gao

**Affiliations:** 1 Tianjin Institute of Industrial Biotechnology, Chinese Academy of Sciences, National Technology Innovation Center of Synthetic Biology, No. 32, Xiqi Road, Tianjin Airport Economic Park, Tianjin, 300308 China; 2 Zhang Zhongjing College of Chinese Medicine, Nanyang Institute of Technology, Nanyang, China; 3 Tianjin Key Laboratory of Animal Molecular Breeding and Biotechnology, Tianjin Livestock and Poultry Health Breeding Technology Engineering Center, Institute of Animal Science and Veterinary, Tianjin Academy of Agricultural Sciences, Tianjin, 300381 China

**Keywords:** *Bacillus subtilis*, Gut microbiota, Mouse evaluation, Nattokinase, Recombinant expression

## Abstract

**Background:**

Nattokinase (NK), a serine protease renowned for its thrombolytic activity, holds immense promise for addressing cardiovascular diseases and metabolic disorders. However, its clinical and industrial translation has been severely hampered by low yields in conventional expression systems, which rely on inefficient solid-state fermentation or suboptimal recombinant platforms. Meanwhile, the global burden of obesity and its associated cardiovascular diseases remain prominent. To this end, implementing a fed-batch fermentation strategy for precise nutrient control is a critical approach to breaking through yield bottlenecks and achieving high-density NK production. Meanwhile, introducing in vivo animal model studies enables a more systematic evaluation of the actual therapeutic efficacy and mechanisms of recombinant NK in improving lipid metabolism and alleviating cardiovascular complications.

**Results:**

First, we engineered a robust recombinant *Bacillus subtilis* strain (T3) by harnessing a strong constitutive promoter (*Pspovg*) and a dual-reporter system (NK-eGFP fusion), enabling real-time monitoring of protein expression. This platform achieved a groundbreaking NK activity of 1.18 × 10^5^ U/mL in shake-flask cultures, surpassing traditional fermentation methods by over threefold. Second, through fed-batch optimization in a 5-L bioreactor, we developed a scalable production protocol that delivered a peak NK activity of 4.19 × 10^5^ U/mL, marking a 61% enhancement over wild-type strains and setting a new paradigm for cost-effective industrial-scale manufacturing. Third, comprehensive animal studies revealed NK’s previously unappreciated metabolic versatility: dietary supplementation (10,000 U/kg BW) in high-fat diet-fed mice not only attenuated obesity-related phenotypes including body weight gain, adiposity, and dyslipidemia, but also restored gut microbiota homeostasis by reversing the dysregulated Bacteroidetes/Firmicutes ratio and enriching taxa implicated in metabolic health.

**Conclusion:**

These findings collectively establish NK as a multifunctional enzyme with applications extending beyond its well-known thrombolytic properties to include metabolic syndrome management. The study provides both a theoretical foundation and practical methodology for large-scale NK production while expanding the therapeutic potential in obesity management. By integrating advances in genetic engineering, fermentation technology, and physiological evaluation, this work represents a significant step forward in harnessing microbial enzymes for therapeutic purposes.

## Introduction

With advances in synthetic biology, food production by microbes is considered to be a promising alternative that would allow rapid food production in an environmentally friendly manner. Moreover, synthetic biology can be adopted to the production of healthier or specifically designed food ingredients (e.g., high-value proteins, lipids, and vitamins) and broaden the utilization of feedstocks (e.g., methanol and CO_2_), thereby offering potential solutions to high-quality food and the greenhouse effect [1].

The fermented soybean product natto is the main source for obtaining purified nattokinase (NK, EC 3.4.21.62), while similar enzymes have also been extracted from other fermented soybean-based foods such as Thai thua nao, Chinese douchi, and Korean doen-jang [2]. NK is encoded by the *aprN* gene with an 1,146 bp open reading frame, translating into a 381-amino acid precursor. This precursor comprises a signal peptide, a pro-peptide, and a mature peptide. The signal peptide guides NK across the cell membrane via the Sec-dependent secretion pathway and the pro-peptide assists in the correct folding of the enzyme [3, 4]. Mature NK is a serine protease composed of 275 amino acids, with an isoelectric point (PI) of 8.6 and an average molecular weight of 27.7 kDa [5]. NK maintains high activity between pH 5.5 and 9.0. When the alkaline pH and temperature are higher than 60 °C, it will affect the structure and function of the enzyme, leading to a decrease in enzyme activity. When the pH is less than 3, NK rapidly becomes inactive [6, 7]. Its three-dimensional structure is highly homologous to subtilisin E, with only differences at two amino acid sites [8].

Conventional production of NK primarily relies on solid-state fermentation (SSF) using substrates such as soybeans [9, 10]. While effective on a small scale, this traditional method presents significant drawbacks for industrial-scale applications, including high product purification costs, limited scalability, and the generation of a pungent odor during the fermentation process [11]. Submerged fermentation (SmF) offers a potential solution to these limitations, providing a more controlled environment suitable for large-scale production. However, a major challenge persists, as the NK enzyme activity yielded by standard SmF is often considerably low [12]. To overcome this bottleneck, researchers have explored heterologous expression of the *aprN* gene, which encodes for NK in alternative microbial hosts such as *Lactobacillus lactis* and *Pichia pastoris* [13, 14]. Despite these efforts, the enzymatic activity of heterologously expressed NK in these systems remains suboptimal, thereby hindering their transition to widespread industrial use.

In this study, *Bacillus subtilis* was selected as the expression host to achieve high-yield NK production. This Gram-positive bacterium is recognized as a safe industrial microorganism due to its absence of endotoxins and exotoxins [15]. Furthermore, *B. subtilis* is well-established as a premier cell factory for the secretion of various industrial enzymes, including proteases, amylases, and lipases, boasting efficient protein secretion pathways and well-characterized genetic tools [16, 17]. The core experimental strategy involved the overexpression of the *aprN* gene in this optimized host.

Driven by the compelling enzymatic properties of NK, we constructed a systematic genetic engineering approach to enhance its production yield. A key tactic was the employment of the strong, constitutive promoter Pspovg to drive high-level transcription of the *aprN* gene. To facilitate rapid and efficient screening of high-performing clones, the target gene was fused with a green fluorescent protein (GFP) tag. This fusion construct enabled the establishment of a high-throughput screening method using flow cytometry, allowing for the precise selection of transformants with superior expression levels. The genetic optimization was seamlessly integrated with an advanced fermentation strategy. By employing high-density fermentation in a controlled 5-L bioreactor, we achieved efficient and scalable NK production in *B. subtilis*, thereby paving the way for large-scale, cost-effective manufacturing.

NK is considered to be a safe, powerful, low cost, and all-natural supplement for the treatment of heart and cardiovascular disease. Obesity is a prominent global health issue, where abnormal fat accumulation caused by the interaction of multiple factors disrupts the balance between energy expenditure and expenditure [18]. obesity is a powerful predictor of sudden cardiac death, which is closely related to the incidence rate and mortality of cardiovascular disease [19]. Currently, over 600 million adults worldwide are affected by obesity (BMI ≥ 30), with projections estimating that this number will reach 4 billion by 2035 [20]. Consequently, the development of effective preventive and therapeutic strategies is imperative to address the global obesity epidemic. Therefore, beyond production optimization, this research also investigated the physiological impact of NK. We evaluated the therapeutic potential of the enzyme by supplementing it into the diet of mice and conducting a comprehensive analysis of its effects on various physiological parameters, including body weight, serum lipid profiles, and gut microbiota composition. These investigations provide a crucial theoretical foundation for the potential applications of NK in the medical field and offer significant support for the future development of related therapeutic technologies.

## Materials and methods

### Bacterial strains and growth conditions

*E. coli* DH5α purchased from Beijing Tsingke Biotech Co., Ltd., was used for plasmid construction and amplification. Expression was performed using *B. subtilis* X16 stored in the laboratory as host cells. Unless otherwise specified, culture in LB medium containing 10 g/L peptone, 10 g/L sodium chloride, and 5 g/L yeast powder. Transformant LB medium supplemented with 100 mg/L Ampicillin was used to select transformants.

*Bacillus subtilis* 5 L fermentation medium: glucose 20 g/L, peptone 25 g/L, NaCl 10 g/L, KH_2_PO_4_ 1 g/L, MgSO_4_ 0.3 g/L, CaCl_2_ 0.3 g/L, Tween-80 0.2 g/L, defoamer 0.2 g/L. Supplements: glucose 180 g/L, peptone 54 g/L, MgSO_4_ 0.3 g/L, CaCl_2_ 0.3 g/L, Tween-80 0.1 g/L.

### Plasmid and strain construction

The promoter P*spovg* fragment was amplified from the *B. subtilis* 168 genome and the fragment of *aprN* gene was amplified from the *B. subtilis* X16 genome. The plasmid vector pHT43-SUMO-eGFP was purchased from Wuhan Miaoling Biotechnology Co., Ltd. The expression plasmid pHT43-P*spovg*-NK-eGFP was constructed via Gibson assembly, and amplification in *E. coli* DH5α. The plasmid was transformed into the chassis cell *B. subtilis* X16 using chemical transformation. Transformants were screened using a flow cytometer based on fluorescence intensity and further verified by gene sequencing and fluorescence microscopy. The strain, plasmid and primers used in this study are shown in Tables 1 and 2.

**Table 1:** Primers and sequences

Primer	Sequence (5′→3′)
P*spovg*-f	actcacattaattgcgttgcgctaagaaaagtgattctgggagagccgggat
P*spovg*-r	tgatccttcctccttaaattgagtagttcaccaccttttccct
NK-X16-f	caatttaaggaggaaggatcaatgagaagcaaaaaattgtggatcagct
RBS-r	tgatccttcctccttaaattgttattgtgcagctgcttgtacgt
RBS-f	caatttaaggaggaaggatcaatgggctctcttcaagattctgaagt
P1-r	gcgcaacgcaattaatgtgagt
P2-f	gtatagcttccacccaagttagcctttctgcttcttctgaatg
P3-r	ctaacttgggtggaagctataccatatcatatgctacgttacattgactttagcgaccct
M13-yz-f	gtaaaacgacggccagt
Pyz-r	ggttaacaagtgtatcgcct

**Table 2:** Strains and plasmids used in this study

Strain	Source
*B. subtilis* X16	Laboratory storage
*B. subtilis* X16-T1	This study
*B. subtilis* X16-T2	This study
*B. subtilis* X16-T3	This study
*B. subtilis* X16-T4	This study
*B. subtilis* X16-T5	This study
*B. subtilis* X16-T6	This study
*B. subtilis* X16-T7	This study
*B. subtilis* X16-T8	This study
*E. coli* DH5α	Beijing Tsingke Biotech Co., Ltd.
Plasmid	
pHT43-SUMO-EGFP	Wuhan Miaoling Biotechnology Co., Ltd.
pHT43-P*spovg* -NK-SUMO-EGFP	This study

### Preparation and transformation method of *B. subtilis* receptive state

A freshly activated single colony was selected and transferred into a 5-mL GMI broth tube. The culture was incubated at 37 °C and 200 r/min for 14–16 h. Subsequently, 500 μL of the culture was transferred to a 4.5-mL GMI broth tube and incubated at 37 °C and 200 r/min for 4.5 h. Following this, 750 μL of the GMI culture was transferred to 4.25 mL GMII broth and incubated at 37 °C and 240 r/min for 1.5 h. Finally, competent cells were prepared by dispensing aliquots into 1.5-mL centrifuge tubes. One to two micrograms of donor DNA was added to 1 mL of competent cells, and the mixture was shaking cultured at 200 r/min for 12 h. The transformants were then screened by flow cytometry based on their fluorescence intensity, and the top 0.2% of transformants with fluorescence were sorted and cultured on LB plates containing ampicillin for overnight culture.

### Determination of enzyme activity

NK activity was measured spectrophotometrically by using chromogenic substrates. The reaction mixture (1 mL) contained 20 μL of enzyme solution, 5 × 10^−4^ mol/L chromogenic substrate (succinyl-Ala-Ala-Pro-Phe-*p*-nitroanilide), and 0.1 mol/L Tris–HCl CaCl_2_ buffer (pH 8). After incubation for 5 min at 37 °C, the amount of liberated *p*-nitroaniline was determined by the spectrophotometric absorption at 405 nm. An enzyme activity unit (U) is defined as the amount of enzyme that catalyzes the hydrolysis of a tetrapeptide substrate, producing 1 μmol of *p*-nitroaniline per minute under standard assay conditions [21, 22].

### Fed-batch fermentation

A 5-L bioreactor (Baoxing, China) with a working volume of 3 L was employed for the fermentation process. The initial seed culture was prepared in LB medium and incubated at 37 °C until the optical density (OD) reached 2. An inoculation volume of 2% (V/V) was used. The initial stirring speed was set to 200 r/min, and the air flux was set to 1.5 vvm. After inoculation, the air-cascade and speed-cascade controls were activated. When the dissolved oxygen (DO) value fell below 40%, the air flux and stirring speed were automatically increased. The air flux range was set to 1.5–7.5 vvm, and the stirring speed range was set to 200–800 r/min. Optimal pH (7.2) and temperature (37 °C) were maintained in the bioreactor throughout the exponential growth phase. Feeding was initiated when the substrate in the basal medium was depleted, as indicated by the significant increase in DO concentration. During the feeding phase, the DO level was regulated to maintain a range between 20% and 30% by adjusting the feeding rate. At two-hour intervals, samples were withdrawn to assess both OD and enzyme activity.

### Analysis of specific growth rate and specific enzyme production rate

The specific growth rate (*μ*) was determined using optical density at 600 nm (OD_600_) measurements during the exponential growth phase. The specific enzyme production rate (*q*_*p*_) was calculated based on the change in extracellular nattokinase activity over the same interval. The parameters during the exponential phase can be quantified using the formula:

1 $$\mu=\frac{\mathrm{ln}({\mathrm{OD}}_2)-\mathrm{ln}({\mathrm{OD}}_1)}{(t_2-t_1)}$$

2 $${q}_{p} =\frac{\Delta {Enzyme\ Activity}}{\Delta Biomass\times\Delta t}$$

where OD_1_ and OD_2_ represent biomass concentrations at times *t*_1_ and *t*_2_, respectively. The change in enzyme activity (ΔEnzyme Activity) was defined as the difference in activity between *t*_2_ and *t*_1_, while the change in biomass (ΔBiomass) corresponded to the difference in OD_600_ values.

### Animal models, diet, and sample collection

Specific pathogen-free (SPF) grade male C57BL6J mice aged at 7 weeks were purchased from Vital River Laboratory Animal Technology Co., Ltd. (Beijing, China). All mice were housed and kept in an SPF animal experimental center (temperature, 22 ± 2 °C; relative humidity, 50% ± 10%; light, 12 h light/dark cycle) with free access to feed and water. All animal procedures were conducted following the Guidelines of the Laboratory Animal Ethics Committee and were approved by the Animal Care and Use Committee of Nanyang Institute of Technology, Chinese Academy of Sciences (Protocol Approval Number: 2023-064). After one-week acclimatization, the mice were randomly divided into three groups as follows: a control (CON) group (*n* = 8) fed chow diet (D12450B, Research Diets, Inc.) and gavaged with normal saline; a high fat diet (HFD) group (*n* = 8) fed high fat diet (HFD, D12492, Research Diets, Inc., New Brunswick, NJ, USA) and gavaged with normal saline; a HFD + NK group (*n* = 8) fed HFD and gavaged with NK (dissolved in normal saline) at a dose of 10,000 U/kg BW. The mice trial lasted for 52 d. At the end of the trial, all mice were deeply anesthetized with isoflurane to collect blood from the eye socket after 12 h of fasting, then sacrificed by cervical dislocation. The serum was obtained by centrifugation at 3,000 × *g* for 15 min at 4 °C and then stored at −80 °C. The liver, spleen, kidney, heart, epididymal and perirenal white adipose tissues (WATs) were manually isolated from mice and weighted with analytical balance rapidly. Fecal samples were collected and immediately transferred into liquid nitrogen or −80 °C for the microbiota analysis.

### Hematoxylin & Eosin (H&E) staining and histopathological analysis

The liver and epididymal WAT samples from mice were fixed in 4% paraformaldehyde, followed by gradient dehydration, paraffin infiltration, and embedding. The embedded tissues were then sectioned into 4 μm thick paraffin slices using a microtome. After completing deparaffinization and rehydration procedures, the tissue sections were stained using the H&E method. Briefly, sections were first deparaffinized in xylene. Rehydration was performed using a graded ethanol series (100%, 95%, 85%, and 75%), with each concentration applied for 5 min, followed by a tap water rinse. The samples were stained with hematoxylin for 2 min and then rinsed under running water for 15 min. Next, eosin staining was carried out for 30 s, followed by a 5-min running water rinse. Dehydration was achieved via the reverse sequence of the graded ethanol series, and the sections were cleared with xylene prior to mounting with neutral resin. After air drying, the sections were scanned by Olympus microscope (Markham, ON, Canada).

### Serum biochemical analysis

The serum samples taken out from −80 °C refrigerator were thawed at 4 °C. An Automated Biochemistry Analyzer (Bokang Biotechnology Co., Ltd., Shandong, China) and the corresponding detection kits were used to determine the triglycerides (TG) and total cholesterol (TC) concentrations, with all operations conducted in accordance with the manufacturer’s recommended protocols (Bokang Biotechnology Co., Ltd., Shandong, China).

### Oral glucose tolerance test (OGTT)

On the 42^nd^ day following an overnight fast, the mice were weighed. Subsequently, all mice received 2 g/kg BW glucose by oral gavage, and blood samples was collected from the tail vein of mice and determined by blood glucose test strips (Roche Diagnostics Co., Ltd.) and blood glucose meter (Roche Diagnostics Co., Ltd.) at 0, 30, 60, 90, and 120 min. The glucose concentration curve against the time for OGTT was plotted and the areas under the curve (AUC) was also calculated and plotted.

### Gut microbiota analysis

Fecal bacterial DNA in mice of each group was extraction using the QIA amp Fast DNA Stool Mini Kit (QIAGEN, Dusseldorf, Germany) according to the protocols of the manufacturer. The Nanodrop ND-1000 spectrophotometer (Nanodrop Technologies, Delaware, USA) was used to quantify the concentration and purity of bacterial DNA from each sample. Meanwhile, the integrity of the extracted DNA through 1% agarose gel electrophoresis. For amplification of the V3–V4 regions in the bacterial 16S rRNA gene, universal primers-capable of targeting this region across most bacterial species were employed. The specific primers used were 338 F (5′-ACTCCTACGGGAGGCAGCA-3′) and 806R (5′-GGACTACHVGGGTWTCTAAT-3′). After PCR amplification, the purified amplicons were connected to sequencing adapters to build sequencing libraries. These libraries were then sequenced on the Illumina NovaSeq platform (Novogene, Beijing, China). All 16S rRNA sequences were OTU clustered using UPARSE software based on their 97% similarity. Rarefaction curves were applied to assess the sufficiency of sample coverage and the level of species richness. Alpha diversity analysis was performed to assess the diversity within individual microbial community samples using the Simpson Wilcox, Dominance, Chao 1, and Shannon index. The β diversity analysis was conducted to compare species diversity across the different samples, considering both community composition and structure using principal component analysis (PCA). The abundance heatmap illustrated similarities and differences in composition between the samples and groups. The significant differences between groups were assessed using linear discriminant analysis effect size (LEfSe) and analysis of variance. LEfSe was employed to identify key bacteria (ranging from phylum to species) that were differentially represented, with a linear discriminant analysis (LDA) significance threshold set at ≥ 4.0. Spearman correlation heatmap analysis was used to evaluate the relationship between obesity-related indicators and gut microbiota.

### Statistical analysis

Statistical analyses were performed via one-way analysis of variance (ANOVA) with Duncan multiple range post hoc test, using SPSS Statistics version 20 (IBM Corp., Armonk, NY, USA). Values were presented as the mean ± standard error of the mean (SEM) or total SEM. *P* < 0.05 was considered statistically significant among different groups. The bar graphs were generated using GraphPad Prism version 10 (Boston, USA).

## Results

### Recombinant expression of NK in *B. subtilis*

**Fig. 1:**
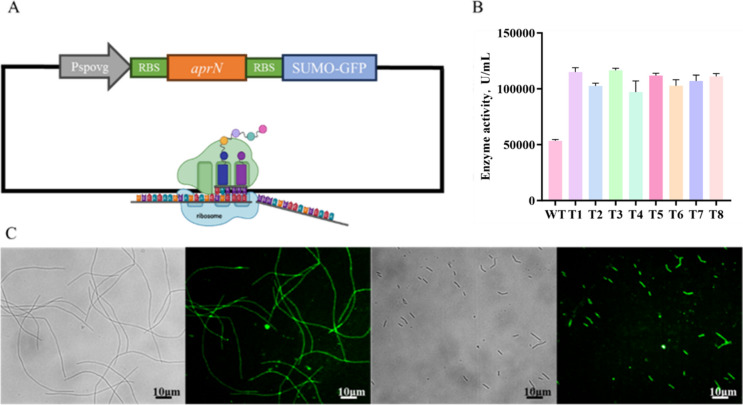
Genetic engineering of *B. subtilis* for NK production.** A** Schematic illustration of the constructed vector pHT43-Pspovg-NK-eGFP in this study. **B** Comparison of NK activity between wild-type and transformant strains. **C** Fluorescence microscopy imaging of transformant T3 cultured in LB medium for 6 h (Left) and 18 h (Right). Scale bar: 10 μm

To achieve efficient recombinant expression of NK, we constructed the recombinant plasmid pHT43-Pspovg-NK-eGFP. As schematically illustrated in Fig. 1A, this plasmid comprises the strong constitutive promoter Pspovg, the target gene *aprN* encoding NK, and an enhanced green fluorescent protein (eGFP) gene serving as a fluorescent reporter. The target gene and the reporter gene are independently linked by a ribosome binding site (RBS) sequence to ensure simultaneous translation. The constructed plasmid was subsequently introduced into competent *B. subtilis* X16 cells via chemical transformation. Initial screening of transformants, was performed using flow cytometry to isolate clones exhibiting enhanced fluorescence signals, indicative of successful plasmid uptake and robust expression. From this pool, eight transformants with the highest fluorescence intensity, along with the wild-type strain, were selected for further evaluation. These strains were cultivated in 50-mL shake flasks to compare their NK production capabilities.

The enzyme activity assay revealed that all selected transformants exhibited significantly higher NK activity compared to the wild-type *B. subtilis* X16 (WT) strain. Quantitative analysis, presented in Fig. 1B, identified transformant T3 as the highest producer, achieving a remarkable extracellular NK activity of 1.18 × 10^5^ U/mL in the shake-flask culture. Although the genetic composition among different transformants is identical, plasmid copy number is regulated by the origin of replication, and the number of plasmids per cell is influenced by various factors including growth conditions, temperature, and other extracellular stresses. However, excessive copy numbers can also impose a heavy metabolic burden on the host, thereby reducing population fitness [23]. Meanwhile, there is heterogeneity in gene expression among cells. Even when the cellular state within a population is uniform, stochastic effects can cause variations in the timing and order of molecular processes governing transcription and translation in individual cells [24]. The average activity of T1 and T3 was similar, but T3 showed slightly higher activity than T1 across three independent replicate experiments. Additionally, T3 exhibited lower batch-to-batch variation. Therefore, T3 was selected as the representative strain for subsequent experiments. This result confirms the success of the overexpression strategy. The strong fluorescence observed in transformant T3 under microscopy (Fig. 1C) further verified the stable maintenance and expression of the plasmid within the *B. subtilis* X16 chassis cells. It is worth noting that the cellular morphology of *B. subtilis* varied with growth phases. During the exponential phase, cells tended to align in linear chains, while in the stationary phase, they dispersed into single cells or small clusters, concomitant with the initiation of sporulation, as visible in the 18 h image. This successful genetic engineering and screening process established a solid foundation for the subsequent scale-up fermentation.

### High-density fermentation of recombinant *B. subtilis* X16 for efficient production of NK

To scale up the production of NK and evaluate its industrial potential, we performed high-cell-density fermentation using the recombinant strain *B. subtilis* T3 in a 5-L bioreactor with a working volume of 3 L. The fermentation process was conducted under tightly controlled conditions, with temperature maintained at 37 °C and the initial pH set at 7.2. The inoculum size was 2% (v/v). We systematically monitored key process parameters-including dissolved oxygen (DO), biomass accumulation (measured as optical density, OD), and NK enzyme activity, at regular intervals throughout the fermentation to assess kinetic profiles and production efficiency.

During the initial 12 h, the cells entered a rapid exponential growth phase, consuming significant amounts of oxygen and resulting in a sharp decline in DO levels. This stage was predominantly dedicated to biomass formation, with only minimal NK activity detected. Upon depletion of the initial carbon source, indicated by a sudden rebound in DO concentration, a fed-batch strategy was initiated. The feeding rate was dynamically adjusted to maintain DO levels within an optimal range of 20%−30%, ensuring adequate aeration and nutrient supply while preventing oxygen limitation or overflow metabolism.

**Fig. 2:**
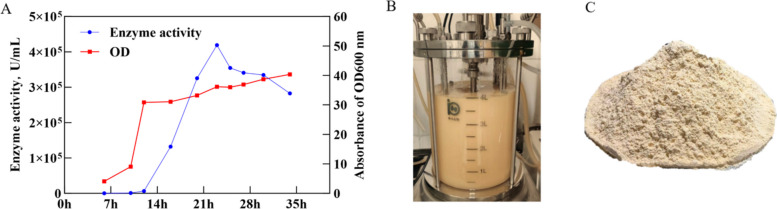
High-density fermentation profile of NK production.** A** High-density fermentation results of the transformed product. **B** Image of 5-L bioreactor. **C** Freeze drying after starch embedding

As cell growth stabilized and entered the stationary phase, a substantial increase in NK production was observed, indicating a transition from growth-associated to production-phase metabolism. Enzyme activity rose markedly, culminating in a peak NK yield of 4.19 × 10^5^ U/mL at the 23 h mark (Fig. 2A and B). Generally, in *Bacillus subtilis*, most protein secretion occurs at the early stationary phase of growth. Protein secretion activity is relatively low during the exponential phase but increases significantly at the onset of the stationary phase, which is consistent with the trend shown in Fig. 2A [25]. On the other hand, bacterial secretion stress response may also affect extracellular product levels [26]. The specific growth rate (*μ*) during the exponential phase was calculated as 0.61 h^−^^1 ^using OD_600_ values at two consecutive time points (OD_1_ = 9.05 at *t*_1_ = 10 h; OD_2_ = 30.85 at *t*_2_ = 12 h), confirming robust biomass accumulation under tight substrate regulation via the bioreactor’s fed-batch system. Concurrently, the specific enzyme production rate (*q*_*p*_) was determined as 119.5 U/OD/h, derived from the change in enzyme activity (ΔEnzyme Activity = 6,290 U/mL − 1,079 U/mL = 5,211 U/mL) and biomass (ΔBiomass = 30.85 − 9.05 = 21.80 OD units) over the same interval. This high-density fermentation strategy markedly enhanced both volumetric productivity and process economy compared to shake-flask cultures [27].

Prior studies achieved maximum NK activities of 18,014 IU/mL [28], 9 × 10^4^ IU/mL [29], 390 FU/g [30], or 425.00 FU/g [30]. Our results show that the recombinant strain T3 achieves 4.19 × 10^5^ U/mL NK activity in a 5-L bioreactor under fed-batch conditions, which represented a 61% improvement over wild-type strains and exceeding most reported values in the literature. Even at the shake-flask level, our system delivers 1.18 × 10^5^/mL, outperforming conventional solid-state and submerged fermentation approaches. Collectively, these results established our study as a more economically viable option for industrial NK manufacturing (Table 3).

**Table 3:** Comparative overview of nattokinase production performances

Strain/system	Fermentation mode	NK activity	Reference
Engineered *B. subtilis* T3	Fed-batch	4.19 × 10^5 ^U/mL	This study
*B. subtilis* JZ08-02	Fed-batch	18,014 IU/mL	[28]
*B. subtilis* BSNK-5	Soybean whey fermentation	9 × 10^4^ IU/mL	[29]
Co-fermentation (*B. natto* + *Bifidobacterium*)	Potato-soy fermentation	390 FU/g	[30]
Coix seed natto co-fermentation	Solid-state	425 FU/g	[30]

Following fermentation, the culture broth was centrifuged to separate the cell-free supernatant, which contained the secreted NK. The supernatant was subsequently mixed with edible starch as a stabilizer, followed by freeze-drying to produce a solid formulation (Fig. 2C) with an activity of 2 × 10^5^ U/g. This powdered preparation was stored under stable conditions for use in subsequent animal feeding trials and further functional evaluations. The successful scale-up demonstrates the robustness of the engineered strain and the effectiveness of the integrated fermentation strategy for achieving high-yield, cost-effective production of NK.

### Effect of NK on body weight-related parameters, serum lipid profiles, and oral glucose tolerance in HFD fed mice

**Fig. 3:**
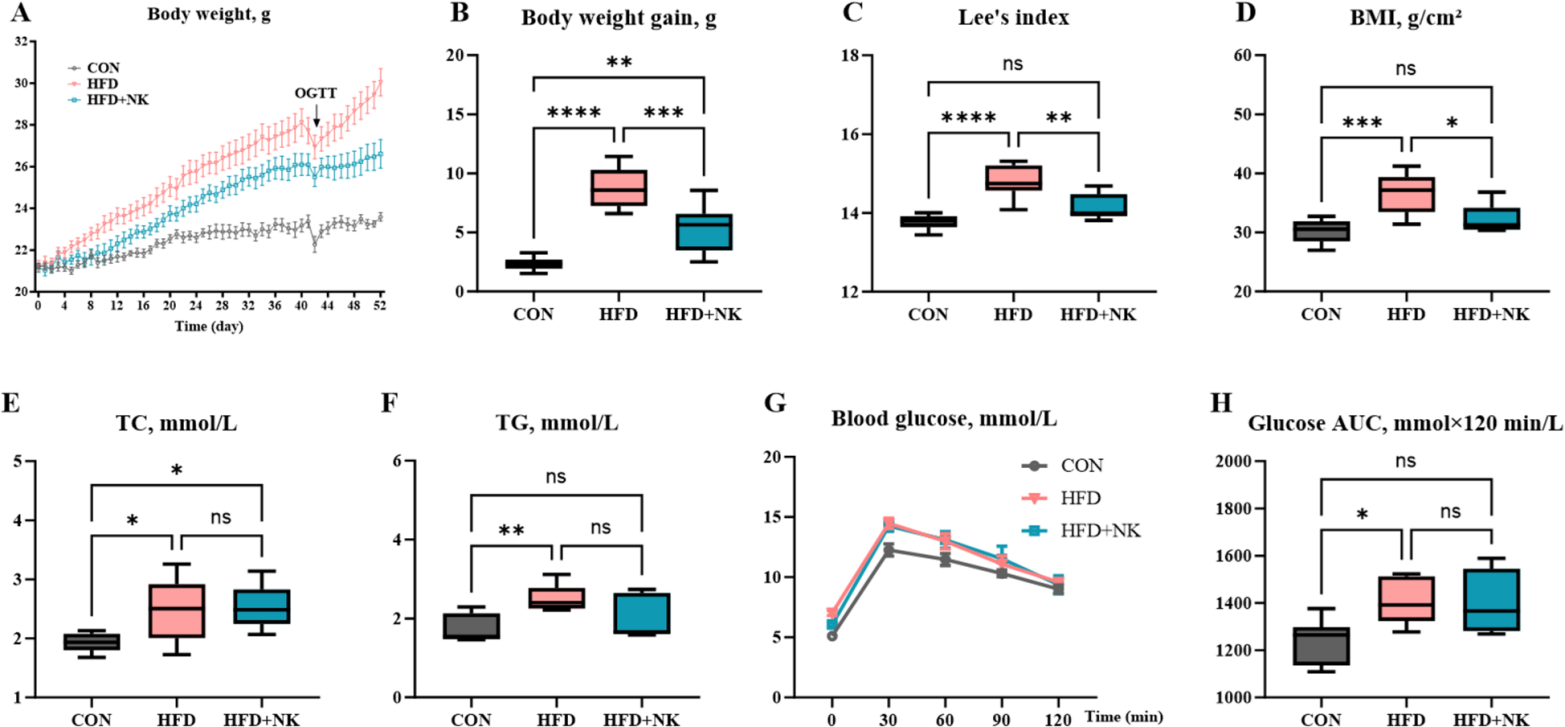
Body weight-related parameters, serum lipid profiles, and oral glucose tolerance in mice (*n* = 6–8). **A** Dynamic changes in body weight throughout the experimental period (*n* = 8). **B** Body weight gain of mice. **C** BMI index. **D** Lee’s index. **E** Serum total cholesterol level. **F** Serum triglyceride level. **G** Oral glucose tolerance test (OGTT) curve. **H** Area under the curve (AUC) of blood glucose in the OGTT. The data were expressed as mean ± SEM. ^*^P < 0.05; **P < 0.01; ***P < 0.001; ****P < 0.0001; ns, not significant

The body weight of mice was recorded daily during the trial period. As shown in Fig. 3, mice fed with a high-fat diet had greater body weight gain (*P* < 0.05, Fig. 3B), BMI (*P* < 0.05, Fig. 3C) and Lee’s index (*P* < 0.05, Fig. 3D) when compared with those in the CON group, which were the vital obesity indicators. Significantly, NK administration inhibited (*P* < 0.05) these increase in the mice fed with a high-fat diet. Both serum TC and TG of mice are shown in Fig. 3E and F. Actually, the serum TC (*P* < 0.05) and TG (*P* < 0.05) in the mice were induced by a high-fat diet when compared to those in the CON group, and NK supplement alleviated the TG increase in the mice from HFD + NK group. Blood glucose and ACU value determined by OGTT are key indexes that is associated with the obesity-related effects. As shown in Fig. 3G and H, compared to the CON group, high-fat diet led to a raise in the AUC of blood glucose (*P* < 0.05), while NK intervention did not affect the glucose tolerance of high-fat diet mice (*P* > 0.05).

### Effects of NK on organ weight and index in high-fat diet mice

**Fig. 4:**
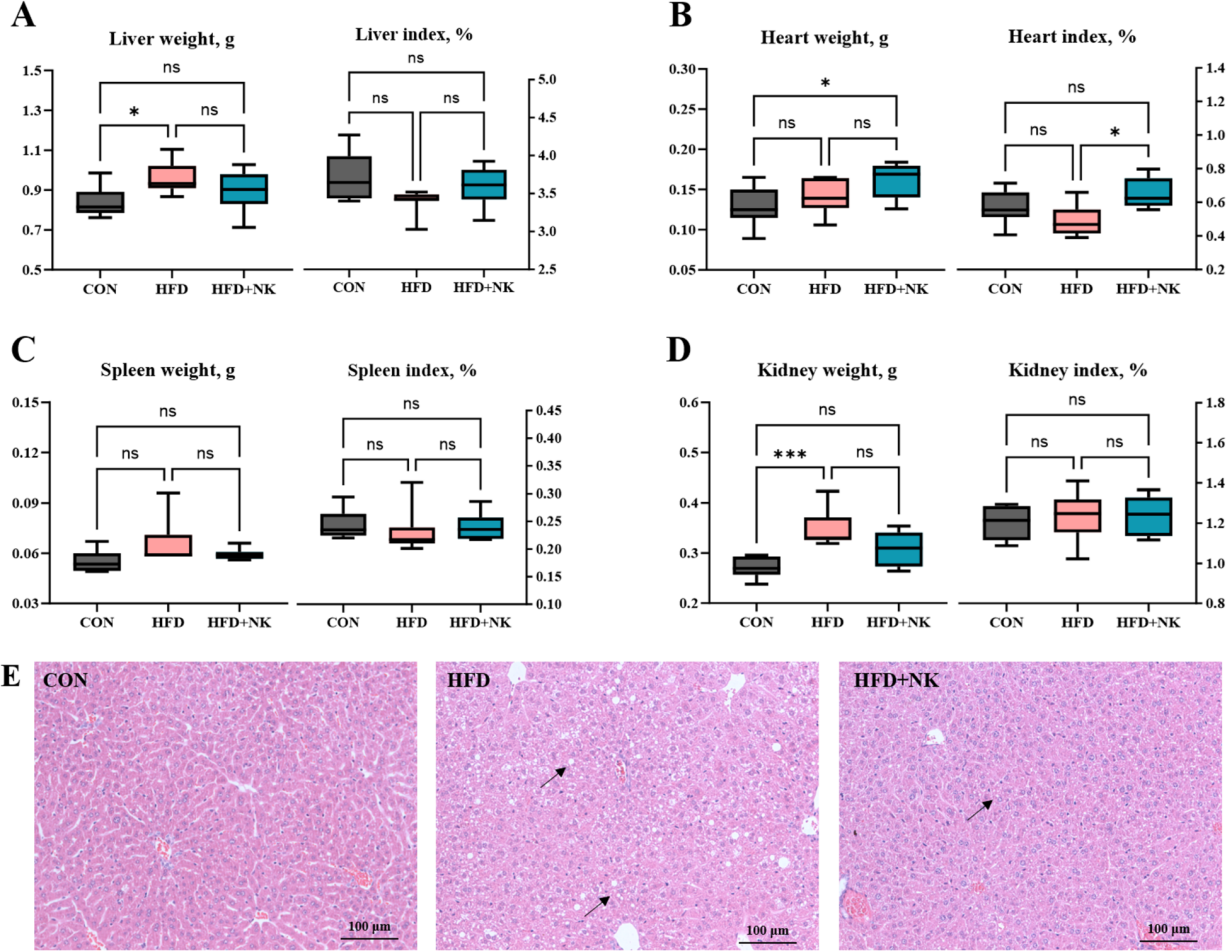
The organ weight and index of mice (*n* = 6–8). **A** Liver weight and index. **B** Heart weight and index. **C** Spleen weight and index. **D** Kidney weight and index. **E** Representative H&E staining images of liver tissue. The black arrows indicated fatty vacuoles. The data were expressed as mean ± SEM. ^*^P < 0.05; **P < 0.01; ***P < 0.001; ns, not significant

The weight of the liver, heart, spleen, kidney, and the corresponding organ indexes are shown in Fig. 4. The absolute weight of the liver (*P* < 0.05, Fig. 4A), spleen (*P* < 0.05, Fig. 4C) and kidney (*P* < 0.05, Fig. 4D) increased with the weight of mice from HFD group when compared with those in the CON group, but NK administration alleviated these rises although the kidney weight in the HFD + NK group was still greater (*P* < 0.05) than that in the CON group. In addition, NK also increased the heart weight (*P* < 0.05) and heart index (*P* < 0.05) when compared to the CON group and HFD group, respectively.

Notably, the liver index of mice in the HFD group significantly reduced (*P* < 0.05) although the liver weight increased when compared to the CON group (Fig. 4A), and there was no obvious difference between the CON group and HFD + NK group (*P* > 0.05). Further, H&E staining results showed that high-fat diet promoted the formation of fatty vacuoles (Black arrows) in the liver, which was significantly inhibited by the NK intervention (Fig. 4E).

### NK reduced WAT accumulation in high-fat diet mice

**Fig. 5:**
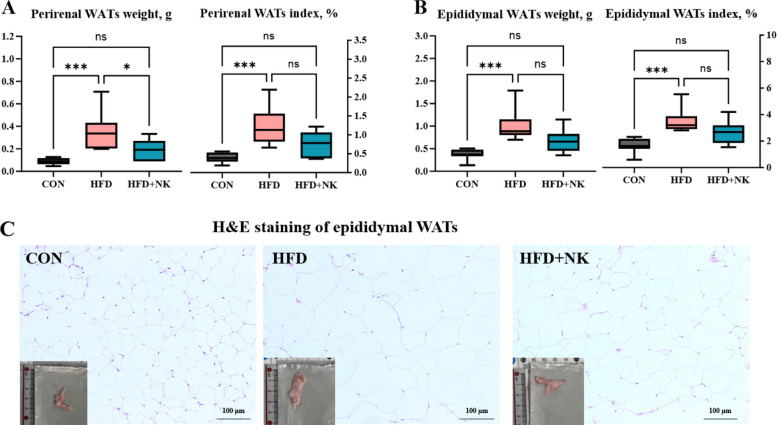
The white adipose tissues accumulation in the mice (*n* = 6–8). **A** Perirenal white adipose tissues weight and index. **B** Epididymal white adipose tissues weight and index. **C** Representative H&E staining images of epididymal white adipose tissues. The data were expressed as mean ± SEM. ^*^P < 0.05; **P < 0.01; ***P < 0.001; ns, not significant

As shown in Fig. 5A and B, the high-fat diet increased the weight of WATs in the mice from HFD group, including perirenal fat (*P* < 0.05) and epididymal fat (*P* < 0.05), when compared with those in the CON group. Indeed, NK significantly reduced (*P* < 0.05) the weight and index of the perirenal fat and epididymal fat in the mice fed with a high-fat diet. In addition, the H&E staining results further demonstrated that NK could inhibit the fat accumulation in the epididymal adipocyte induced by a high-fat diet (Fig. 5C). This phenomenon was consistent with the results of liver tissue H&E staining, which may further explain the anti-obesity effect of NK.

### NK affected the composition and structure of gut microbiota in high-fat diet mice

**Fig. 6:**
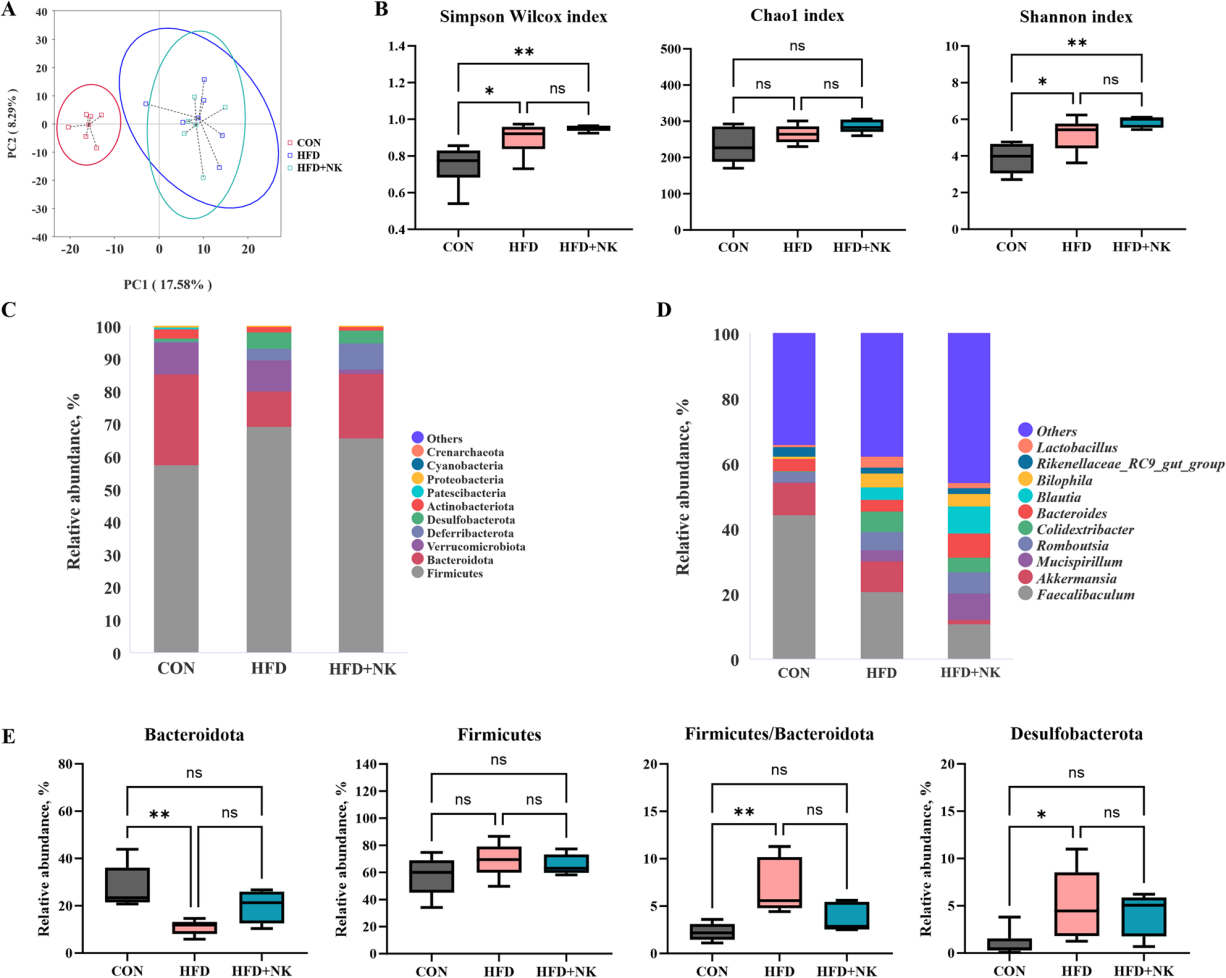
The gut microbial structure analysis in the mice (*n* = 5 or 6). **A** The α diversity indices (Simpson Wilcox index, Chao1 index, and Shannon index). **B** The β diversity analysis: Principal component analysis of gut microbiota. **C** The composition of bacteria at the phylum level. **D** The composition of bacteria at the genus level. **E** Relative abundance of key bacterial taxa at the phylum level. Error bars denote SEM. ^*^P < 0.05; **P < 0.01; ns, not significant

The gut microbiota, a central component of the intestinal microecology and serves as a crucial link between diet and health [31]. The α diversity of gut microbiota was evaluated by the Simpson Wilcox, Chao1, Shannon index, and Dominance (Fig. 6A). Indeed, both Simpson and Shannon indexes of the gut microbiota increased (*P* < 0.05) and the Dominance index reduced (*P* < 0.05) in the mice fed a high-fat diet, indicating that high-fat diet may reduce the total richness and species of gut microbiota, and NK supplement can’t change this variation trend. As presented in Fig. 6B, the gut microbiota was significantly separated (*P* < 0.05) by the high-fat diet and NK according to the Principal Component Analysis (PCA).

The difference of taxonomic profiling of colonic fecal microbiota at different levels among each group were also evaluated in our study. The phylum composition of gut microbiota is presented in Fig. 6C and D. The gut microbiota was predominantly composed of Firmicutes and Bacteroidota at the phylum level, and Firmicutes almost accounted for more than 50% abundance in three groups, especially in the HFD group and HFD + NK group. HFD induced an obvious decrease of the relative abundance of phylum Bacteroidota in the HFD group (*P* < 0.05, Fig. 6E). Particularly, the Firmicutes*/*Bacteroidota ratio and the relative abundance of Desulfobacterota was significantly increased due to the HFD (*P* < 0.05). Actually, NK intervention in the mice fed a high-fat diet didn’t remarkably affect the phylum composition when compared with other two groups (*P* > 0.05).

### Specific phylotypes of fecal microbiota and the correlation analysis between obesity-related indicators and gut microbiota

**Fig. 7:**
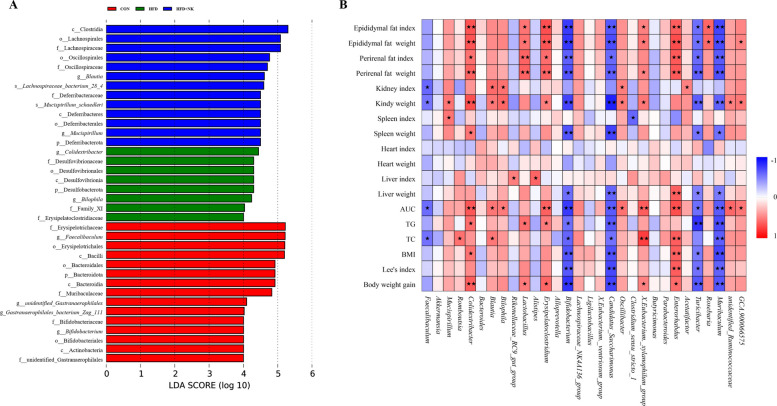
The linear discriminant analysis effect size (LEfSe) analysis and Spearman correlation heatmap of gut microbiota (*n* = 5 or 6). **A** LEfSe analysis identified the significantly different species of gut microbiota among CON group, HFD group, HFD + NK group. **B** Spearman correlation heatmap identified the correlations between the gut microbiota at genus level and obesity-related indicators of each group. Red color indicates positive correlation; blue color means negative correlation. Significant correlations are marked with ^*^P < 0.05 and ^**^P < 0.01

The LEfSe analysis is used to compare multiple groups and identify species with significant abundance differences between different groups. The values of LDA score more than 4 are presented in Fig. 7 to display the significantly enriched species within each group and their influence degrees. Indeed, the mice in CON group were mainly enriched in gut microbiota taxa including the genera *unidentified_Gastranaerophilales*, *Bifidobacterium*, *Faecalibaculum*. In the HFD group, the dominant genera were *Colidextribacter* and *Bilophila*, whereas *Mucispirillum* and *Blautia* were predominant in the HFD + NK group.

To further explore the relationships between the fecal gut microbiota composition changes and the obesity-related parameters among the CON group, the HFD group and HFD + NK groups, the Spearman correlation analysis was performed. As shown in Fig. 7B at genus, some specific gut microbiota mediated by supplementation with NK were found to play a beneficial role in protecting the obese mice against obesity related indices. The *Colidextribacter*, *Lactobacillus*, and *Erysipelatoclostridium* were significantly positively correlated with the perirenal fat weight, perirenal fat index, epididymal fat weight, epididymal fat index, AUC and body weight gain (*P* < 0.05 or *P* < 0.01), indicating that they are the dominant genus leading to obesity-induced WAT accumulation. Meanwhile, *Colidextribacter*, *Lactobacillus*, and *Erysipelatoclostridium* were positively correlated with serum TG (*P* < 0.05 or *P* < 0.01), *Romboutsia*, *Lactobacillus*, *Erysipelatoclostridium* were positively correlated with serum TC (*P* < 0.05 or *P* < 0.01), indicating that they are the dominant genera leading to obesity-induced serum lipid disorder. In contrast, *Bifidobacterium*, *Candidatus_Saccharimonas*, *Turicibacter*, and *Muribaculum* were significantly negatively correlated with perirenal fat weight, perirenal fat index, epididymal fat weight, epididymal fat index, BMI, AUC, Lee's index and body weight gain (*P* < 0.05 or *P* < 0.01), indicating that NK likely improved HFD-induced obesity via enhancing their relative abundance in the feces of obese mice. The *Bifidobacterium*, *Candidatus_Saccharimonas*, *Turicibacter*, and *Muribaculum* were significantly negatively correlated with serum TG (*P* < 0.05 or *P* < 0.01), *Faecalibaculum*, *Bifidobacterium*, *Candidatus_Saccharimonas*, and *Muribaculum* were significantly negatively correlated with serum TC (*P* < 0.05 or *P* < 0.01), indicating that they are the dominant genera leading to obesity-induced serum lipid disorder. These results revealed that NK might improve obesity-related metabolic disorder through modulation of some specific intestinal flora.

## Discussion

NK-producing strains are available from various fermented foods, such as traditional Chinese fermented food bean paste, Indonesian Douchi, Korean Doenjang and Japanese natto [32]. The industrial production of nattokinase mainly relies on strain fermentation, which is divided into solid-state and liquid fermentation. Solid state fermentation materials such as soybeans have low cost and simple technology, making them suitable for small-scale production. However, their yield, detection, and purification are limited. Liquid fermentation uses monosaccharides as carbon sources and peptides and ketones as nitrogen sources, making it difficult to separate and purify the products and producing a lot of waste [33].

In this study, the implementation of genetic engineering and overexpression strategies resulted in a 67% increase in NK activity within a 5-L bench-top fermenter relative to the parental strain. Integrating multifaceted bioengineering strategies is essential for enhancing enzyme activity. Among them, genetic engineering is still the main way to improve activity. Recent advances demonstrate that functional characterization and site-directed mutagenesis of the propeptide (variants Y106V and A103T) can substantially enhance both the folding efficiency and specific activity of the mature protease [34]. Surface charge engineering is equally effective, specifically the substitution of basic and neutral amino acid residues with acidic amino acid residues, has been shown to enhance fibrinolytic activity by modulating the conformational flexibility of regions flanking the active site [35]. In addition to protein engineering, the modification of the host cell chassis, such as Asfandyar et al. [36] through cell membrane engineering, overexpressing *bkdR*/*plsY*/*plsC* genes, and deleting *pssA* and *clsA* genes, has been proven to effectively increase membrane permeability and protein secretion, thereby increasing the yield of heterologous NK. Fermentation process optimization remains equally critical; for instance, Lan et al. employed a co-culture strategy involving *Bacillus subtilis* and Mucor or other probiotics to synergistically elevate NK activity while mitigating the accumulation of biogenic amines [37, 38]. Furthermore, co-production systems for poly-γ-glutamic acid and NK demonstrate that simultaneous metabolic flux without cross-interference can maximize substrate utilization [39]. Conventional culture medium optimization continues to serve as a fundamental supplementary approach to improve NK enzyme activity [40]. Notably, directly comparing enzyme activity values remain challenging due to the assay methods heterogeneity in different studies. Current analytical techniques, including fibrin plate assays, clot lysis time methods, and chromogenic tetrapeptide kinetic assays, each exhibit distinct levels of sensitivity and specificity [32]. Notwithstanding the inherent discrepancies among assay methodologies, the substantial titers achieved in this study underscore the efficacy of an integrated, multi-strategy approach in bridging the gap between bench-scale optimization and industrial-scale manufacturing requirements.

Downstream processing remains a critical bottleneck for the industrial application of NK, necessitating an optimized trade-off among purity, yield, and cost-efficiency. Traditional purification schemes usually involve ammonium sulfate precipitation, followed by multi-step chromatography like ion exchange and gel filtration to achieve electrophoretic homogeneity [41, 42]. While these rigorous methodologies are indispensable for elucidating enzyme kinetics and structural mechanisms, their labor-intensive nature and prohibitive operational costs frequently impede their feasibility for large-scale functional food production. To address scalability challenges, Aqueous Two-Phase Systems (ATPS) have emerged as a promising alternative strategy. Utilizing polyethylene glycol and phosphate salts, ATPS consolidates concentration and partial purification into a single unit operation, offering a cost-effective route for recovering NK with high fibrinolytic activity [43]. However, in the context of dietary supplements, absolute enzymatic purity may be considered subordinate to parameters such as stability and bioavailability. Concurrently, advancements in delivery system design are imperative. Recent research has shown that ovalbumin-flavonoid hydrogels and W/O/W emulsions stabilized by soybean isolate protein and PGA complexes, demonstrate that robust carrier systems can effectively shield NK from gastric degradation and enhance intestinal absorption, thereby obviating the strict requirement for ultra-high purity [44, 45]. Consequently, future trajectories in the food industry will likely prioritize the synergistic integration of simplified downstream processing and efficient encapsulation technologies thereby maximizing economic viability while ensuring therapeutic efficacy.

Natto mucin triggers allergic reactions and symptoms of chronic urticaria by increasing CD203c levels in eosinophils [46]. The gamma PGA in natto mucus is considered an important cause of delayed allergic reactions [47]. Due to the fact that NK is a small molecule protein, protein purification and magnetic microsphere and reverse micelle extraction methods are widely used for purifying NK. However, currently reported purification methods are usually complex or have low NK activity recovery rates, making high-purity NK difficult to obtain, which affects the promotion of NK applications [32]. *B. subtilis* is a Generally Recognized as Safe (GRAS) food-grade microorganism that is widely found in traditional fermented foods and has been available for human consumption for long periods [48]. The *aprN* gene encoding NK has been cloned and recombinantly expressed in various suitable expression systems, including microbes, insects, and plants [49]. Genetically engineered strains typically exhibit higher NK activity and yield. In this study, a strong promoter was employed to drive the expression of the target gene *aprN* encoding NK, achieving high-yield NK production in *B. subtilis*. Compared with traditional fermentation methods, the biosynthetic approach offers advantages such as lower cost, higher efficiency, and sustainability. Additionally, this method facilitates easier product separation and purification, effectively avoiding allergens present in natto components [50, 51].

NK, a protease enzyme produced by *B. subtilis*, has various biological effects such as lipid-lowering activity, antihypertensive, antiplatelet/anticoagulant, and neuroprotective effects [52, 53]. NK has a relatively lower risk of delivery, a larger tolerable dose, and lacks side effects such as gene mutation and chromosomal aberration induction [33]. In the present study, NK was synthesized and investigated its in vivo effects on obesity-related metabolic disorders. The recommended daily dose range for NK is 2,000–4,000 FU to maintain cardiovascular health, and short-term studies have shown that daily doses ≤ 10,000 FU have no serious side effects [54]. Healthy subjects showed a significant peak serum level of NK at approximately 13.3 ± 2.5 h (mean ± standard error) after ingestion of a single daily dose (2,000 FU) of NK [55]. It suggests that NK can be directly measured in human blood after ingestion, and mice supplemented with the dose of 1,000 FU/kg BW NK were safe in this study. Studies on the effect of NK on body weight have demonstrated that male Sprague–Dawley rats treated with 300 mg/kg BW NK (enzyme activity batch: 21,900 FU/g) exhibited a significant reduction in body weight from d 66 to d 87 [54]. Surprisingly, NK supplementation reduced the body weight of HFD-induced obese mice, as well as the accumulation of WATs including perirenal fat and epididymal fat in this study. Currently, research on how NK reduces fat accumulation or alters fat metabolism pathways is limited. Two prevailing theories are widely accepted, one is that NK targets certain key proteins involved in fat metabolism through its proteolytic activity, thereby modifying the pathways of fat metabolism [31], another explanation is that NK may involve in the increase of cerebral blood flow, thus contributing to the decrease of visceral fat mass [56]. Thus, NK may exert weight loss effects by inhibiting WAT accumulation, providing experimental evidence for its application in interventions for obesity and related metabolic disorders. There is growing evidences about the benefits of NK on the modulation of lipid metabolism profile with dyslipidemia. In a study of 76 patients who were supplemented with oral NK at a daily dose of 6,000 FU decreased in TC and TG [57]. In a clinical study, serum TC levels decreased by 6.8% after 8 weeks in patients with primary hypercholesterolemia treated with 4,000 FU NK [58]. In addition, the combination of NK with red yeast rice extract had a hypolipidemic effect for patients with hyperlipidemia, which decreased TG and TC by 15% and 25%, respectively [59]. Our results here were consistent with the previous finding showing that NK were able to decreased serum TG by 18.94% compared with HFD group mice. Related studies have shown that NK may upregulate the mRNA expression of key lipid metabolism genes such as AMPK, PPAR-α, and PPAR-γ in HFD mice, promote cholesterol excretion in the form of bile acids, improve lipid metabolism disorders, and play a role in reducing blood lipids and maintaining energy homeostasis in the body [60]. Additionally, the HFD + NK group showed no significant differences in the weight and index of the liver, spleen, and kidneys compared with CON and HFD groups, which to some extent confirms the biocompatibility of NK at this intervention dose. However, the heart index was increased after NK supplementation compared with HFD group. Obesity usually occurs and develops simultaneously with a variety of chronic metabolic diseases, which will increase the incidence rate and mortality of cardiovascular diseases, leading to arrhythmia, heart failure and sudden cardiac death [61]. Therefore, the observed increase in heart index warrants attention. The adult heart is considered a terminally differentiated organ with limited regenerative capacity [62]. The effect of NK on the heart may be attributed to its ability to directly degrade fibrin, stimulate the release of plasminogen activator to enhance fibrinolytic activity, and regulate the expression of genes related to fat accumulation and lipid metabolism, thereby influencing thrombotic mechanisms [63]. Nevertheless, the specific mechanism still requires further investigation to clarify the potential impact of NK on the cardiovascular system.

The gut microbiota—dubbed the host’s “second genome”—serves as a key regulator of lipid metabolism and energy homeostasis, with Firmicutes, Bacteroidetes, and Proteobacteria being the dominant phyla in the intestinal ecosystem [64]. Accumulative evidence has suggested that obese individuals have differences in their gut microbiota compared to the lean controls, characterized by reduced numbers of Bacteroidetes and the ratio of Bacteroidetes to Firmicutes in the gut [65–67]. Besides, Desulfobacterota is capable of producing lipopolysaccharides (LPS), a substance that can induce inflammatory responses, cause metabolic disorders, and elicit immune stimulation [68]. Notably, a high-fat diet has been found to markedly elevate the abundance of Desulfobacterota in the relevant microecosystem. In the analysis of mouse gut microbiota in this experiment, there were no significant differences in the proportion of Bacteroidetes, Desulfobacterota and the ratio of Firmicutes*/*Bacteroidetes(F/B) between the NK-treated mice and the CON or HFD groups. This may indicate that NK exerts a beneficial effect on ameliorating obesity-induced microbial dysbiosis, which may represent another potential mechanism by which NK alleviates WAT accumulation in obese mice. This is consistent with previous findings that NK may regulate intestinal flora (up-regulating Bacteroides and down-regulating *Shigella*) further improving liver and intestinal function at the gut-liver axis [53]. Notably, *Blautia*, a prevalent intestinal acetic acid-producing bacterium, can potentially suppress insulin signaling and adipocyte fat deposition [69]. *Mucispirillum*, a member of the Deferribacterota phylum, participates in immune modulation and inflammatory reactions associated with a variety of diseases [70]. It can prevent *Salmonella* *enterica* serovar Typhimurium (*S.* Tm) colitis and acts as a key antagonist of this pathogen [71]. *Blautia *and *Mucispirillum*, as enriched microorganisms in the NK group, suggest that these two microorganisms may play an important role in improving metabolic homeostasis caused by a high-fat diet. It may suggest that NK treatment alleviate HFD-induced WAT accumulation and metabolic dysregulation by preserving gut microbiota homeostasis and enriching specific bacteria, including *Blautia* and *Mucispirillum*, which have been associated with various metabolic and immune functions.

## Conclusion

In this study, we successfully constructed an engineered strain for the recombinant expression of NK with the primary objective of enhancing NK activity. Additionally, utilizing an obesity mouse model and gut microbiota analysis, we investigated the potential effects of NK on mouse health and gut microbiota. The results demonstrated that under high-density fermentation conditions, the enzyme activity of transformant T3 reached a maximum of 4.19 × 10^5^ U/mL, surpassing the levels reported in most related studies to date. Evaluation in mice revealed that NK significantly intervened in body weight, effectively regulating weight gain. Furthermore, it exerted positive effects on the organs, fat content, and blood lipid levels in mice, demonstrating favorable physiological regulatory effects. Gut microbiota analysis indicated that NK increased the abundance of gut microbiota in mice and contributed to the restoration of the F/B ratio. These findings provide a crucial theoretical foundation for the production of NK and its potential applications in functional foods and drug development.

## Data Availability

The data that support the findings of this study are available from the corresponding author upon reasonable request.

## Abbreviations

ATPS: Aqueous Two-Phase Systems
AUC: Area under the curve
DO: Dissolved oxygen
eGFP: Enhanced green fluorescent protein
F/B: The ratio of Firmicutes to Bacteroidota
HFD: High fat diet
H&E: Hematoxylin and Eosin
LDA: Linear discriminant analysis
LEfSe: Linear discriminant analysis effect size
NK: Nattokinase
OD: Optical density
OGTT: Oral glucose tolerance test
OTUs: Operational taxonomic units
PCA: Principal Component Analysis
PGA: Poly-γ-glutamic acid
RBS: Ribosome binding site
SmF: Submerged fermentation
SPF: Specific pathogen-free
SSF: Solid-state fermentation
TC: Total cholesterol
TG: Triglycerides
t-PA: Tissue plasminogen activator
WATs: White adipose tissues
WT: Wild-type
